# Dysregulation of post-transcriptional modification by copy number variable microRNAs in schizophrenia with enhanced glycation stress

**DOI:** 10.1038/s41398-021-01460-1

**Published:** 2021-05-28

**Authors:** Akane Yoshikawa, Itaru Kushima, Mitsuhiro Miyashita, Kazuya Toriumi, Kazuhiro Suzuki, Yasue Horiuchi, Hideya Kawaji, Shunya Takizawa, Norio Ozaki, Masanari Itokawa, Makoto Arai

**Affiliations:** 1 Schizophrenia Research Project, Department of Psychiatry and Behavioral Sciences, Tokyo Metropolitan Institute of Medical Science, 2-1-6, Kamikitazawa, Setagaya-ku, 156-8506 Tokyo Japan; 2 Department of Adolescent Psychiatry, Nihonbashi Sun Clinic, 2-1-21, Nihonbashi, Chuou-ku, Tokyo 103-0027 Japan; 3 Department of Psychiatry, Nagoya University Graduate School of Medicine, 65 Tsurumai, Showa-ku, 466-8550 Nagoya, Aichi Japan; 4 Medical Genomics Center, Nagoya University Hospital, Nagoya, Aichi 466-8550 Japan; 5 Department of Psychiatry, Takatsuki Clinic, Tanaka-cho, 196-0014 Akishima, Tokyo Japan; 6 Department of Psychiatry, Tokyo Metropolitan Matsuzawa Hospital, 2-1-1 Kamikitazawa, Setagaya-ku, 156-0057 Tokyo Japan; 7 Research Center for Genome & Medical Sciences, Tokyo Metropolitan Institute of Medical Science, 2-1-6, Kamikitazawa, 156-8506 Setagaya-ku, Tokyo Japan; 8 Division of Neurology, Tokai University School of Medicine, 143 Shimo-Kasuya, 259-1193 Isehara, Kanagawa Japan

**Keywords:** Clinical genetics, Clinical genetics

## Abstract

Previously, we identified a subpopulation of schizophrenia (SCZ) showing increased levels of plasma pentosidine, a marker of glycation and oxidative stress. However, its causative genetic factors remain largely unknown. Recently, it has been suggested that dysregulated posttranslational modification by copy number variable microRNAs (CNV-miRNAs) may contribute to the etiology of SCZ. Here, an integrative genome-wide CNV-miRNA analysis was performed to investigate the etiology of SCZ with accumulated plasma pentosidine (PEN-SCZ). The number of CNV-miRNAs and the gene ontology (GO) in the context of miRNAs within CNVs were compared between PEN-SCZ and non-PEN-SCZ groups. Gene set enrichment analysis of miRNA target genes was further performed to evaluate the pathways affected in PEN-SCZ. We show that miRNAs were significantly enriched within CNVs in the PEN-SCZ versus non-PEN-SCZ groups (*p* = 0.032). Of note, as per GO analysis, the dysregulated neurodevelopmental events in the two groups may have different origins. Additionally, gene set enrichment analysis of miRNA target genes revealed that miRNAs involved in glycation/oxidative stress and synaptic neurotransmission, especially glutamate/GABA receptor signaling, were possibly affected in PEN-SCZ. To the best of our knowledge, this is the first genome-wide CNV-miRNA study suggesting the role of CNV-miRNAs in the etiology of PEN-SCZ, through effects on genes related to glycation/oxidative stress and synaptic function. Our findings provide supportive evidence that glycation/oxidative stress possibly caused by genetic defects related to the posttranscriptional modification may lead to synaptic dysfunction. Therefore, targeting miRNAs may be one of the promising approaches for the treatment of PEN-SCZ.

## Introduction

Schizophrenia (SCZ) is a neurodevelopmental disorder with a worldwide prevalence of around 1.0%, leading to abnormalities in function and connectivity of key brain regions^1^. The most common symptoms are auditory hallucinations, delusions, withdrawal, flat affect, lack of motivation, and disorganized thinking. The onset, during early adolescence, strongly impacts school life and educational achievement, leading to lifelong social and/or occupational dysfunction. Accumulating evidence suggests that both genetic and epigenetic factors are involved in the etiology of SCZ^2–7^. An integrative genetic and epigenetic analysis would accelerate the elucidation of the etiology.

Recent advances in genomic technologies have facilitated large-scale genomic studies. Previous research has successfully uncovered the polygenic nature of multiple common risk variants^4,5,8^ as well as the involvement of rare but highly penetrant variants^9,10^, and structural genetic variants called copy number variations (CNVs) in SCZ^11–15^. In the largest-scale CNV study to date, using 41,321 subjects, deletions in 1q21.1, 2p16.3, 3q29, and 22q11.21 were confirmed as risk factors for SCZ with high odds ratios (ORs) ranging from 3.8–67.7^14^. The Psychiatric Genomics Consortium (PGC)-CNV analysis group has demonstrated that genes disrupted by CNVs in SCZ are enriched within the synaptic networks^14^. Kushima et al. performed the largest genome-wide CNV analysis in the Japanese population so far and confirmed an increased burden of rare exonic CNVs in 2458 SCZ and 1108 autism spectrum disorder (ASD) cases^16^.

MicroRNAs (miRNAs) are a family of short (~22 nt), single-stranded, non-coding RNAs primarily involved in the posttranscriptional downregulation of gene expression via binding to the 3′UTR of target genes^17^. CNV-miRNA genes generate the complexity of miRNA regulation and function^17,18^. Genomic CNVs can induce the aberrant expression of integral miRNAs and their target genes^18^, resulting in the dysregulation of posttranslational modification, crucial for the adaptation to environmental factors. Recently, it has been shown that the genetically dysregulated posttranslational modification caused by CNV-miRNAs contribute to the etiology of neurodevelopmental disorders including SCZ, ASD, and intellectual disability (ID)^19^. For example, in the context of SCZ, miRNA contents within CNV regions were significantly enriched compared to those in control subjects^19^. Additionally, in ASD, miRNAs present in CNV loci were shown to regulate synaptic transmission and may, thus, contribute to the disorder phenotype^20^.

Genetic heterogeneity is a hallmark of SCZ; the genetic architecture of this common disorder is largely complex^21,22^. To overcome the heterogeneity, the identification of SCZ subpopulations presenting with disturbances of specific metabolic pathways is a promising strategy. We previously reported an SCZ subtype with enhanced glycation/oxidative stress that showed accumulated plasma pentosidine (PEN-SCZ)^23^. Although around 20% of SCZ patients were reported to be PEN-SCZ^24^, the genetic and epigenetic factors that account for the accumulation of pentosidine and their contribution to this phenotype have not yet been fully elucidated.

Here, we performed an integrative, genome-wide, CNV-miRNA analysis to investigate the genetic factors associated with SCZ with enhanced glycation/oxidative stress and to provide novel insights into the etiology of PEN-SCZ, focusing on the disturbance of posttranscriptional modification caused by CNV-miRNAs.

## Materials and methods

### Subjects

In this study, 209 unrelated patients with SCZ were recruited, mainly from the Departments of Neuropsychiatry, Tokyo Metropolitan Matsuzawa Hospital, Takatsuki Hospital, Takatsuki Clinic, and RIKEN Brain Science Institute near Tokyo, for both genetic and biochemical analyses. All subjects were ethnically Japanese. The patients were diagnosed according to the DSM-5 criteria (American Psychiatric Association) for SCZ or schizoaffective disorders as per the consensus of at least two experienced psychiatrists. Patients with a history of drug addiction or alcohol abuse/dependence were excluded. Patients with accompanying diabetes mellitus and chronic renal disease were also excluded because these diseases may affect the plasma pentosidine levels. Medical records were obtained with the approval of patients. The study was performed in accordance with the Declaration of Helsinki. This study was approved by the research ethics committee of each participating institute and written informed consent was obtained from all subjects. The subjects’ demographics are presented in Table 1. Of note, to define the PEN-SCZ and non-PEN-SCZ cases, plasma concentration of pentosidine was measured via high-performance liquid chromatography as described previously^25^.

**Table 1:** Demographics and summary of miRNAs within CNVs in schizophrenia patients with/without accumulated plasma pentosidine.

Group	PEN-SCZ^a^	non PEN-SCZ^b^	*P* value/FC^e^
Pentosidine	High	Normal	
Number of patients	94	91	
Age (mean ± S.D.^c^)	52.0 ± 11.2	47.0 ± 14.1	0.0026
Sex (Male/Female)	50/44	46/45	0.68
Ethnicity	Japanese	Japanese	
Age of onset (mean ± S.D.^c^)	25.7 ± 9.4	25.24 ± 8.4	0.37
Plasma pentosidine level (mean ± S.D.^c^) (ng/ml^d^)	128.1 ± 126.0	39.8 ± 9.3	5.19 × 10^−10^
Average length of total CNV (Mb)	3.6 ± 21.9	0.4 ± 0.5	0.083/8.4^e^
Average of chlorpromazine equivalent doses	1147.58 ± 864.02	716.43 ± 607.65	7.33 × 10^−5^
The rate of treatment resistant SCZ (%)	39.3	11.9	0.0022
Average of number of genes within CNVs	92.2 ± 254.2	4.5 ± 7.6	0.08/20.5^e^
Total number of miRNAs within CNVs	205	21	9.8^e^
Number of patients harboring CNV-miRNAs	13 (13.8%)	13 (14.3%)	0.55
Average of the number of miRNAs per patient	15.8	1.6	0.086/9.9^e^
Number of patients with the rate of miRNA genes per total genes within CNVs ≥50%	5	0	0.032
Number of patients with the rate of miRNA genes per total genes within CNVs <50%	89	91	

^a^Schizophrenia with accumulated plasma pentosidine level.

^b^Schizophrenia without accumulated plasma pentosidine level.

^c^Standard deviation.

^d^Cut off value; 55.2.

^e^Fold change.

### Identification of CNV-miRNAs

Genomic DNA was extracted from blood samples. Array comparative genomic hybridization (array CGH) was performed to identify rare (<1%) CNVs in PEN-SCZ and non-PEN-SCZ patients; NimbleGen 720 K Whole-Genome Tiling Arrays (Roche NimbleGen, Madison, WI, USA) were used for the CNV analysis. CNV calls were conducted using the Nexus Copy Number software v9.0 (Bio Discovery, El Segundo, CA, USA) with the Fast Adaptive States Segmentation Technique 2 algorithm. Quality control (QC) was performed as described previously^16,26^. Briefly, QC scores for each sample based on the statistical variance of the probe-to-probe log ratios were calculated and samples with QC >0.15 were removed. As sample QC, subjects with excessive numbers of autosomal CNVs were also removed from the analysis. After filtering out common CNVs (≥1%), rare CNVs (<1%) in 185 patients were used for further analyses. All genomic locations are given in GRCh38 coordinates, and gene annotation was based on GENCODE version 27. Of note, previously, we confirmed that CNV calls from NimbleGen arrays are highly accurate with a validation rate >99%^27^. The miRNAs present in CNVs were then identified via the analysis of the chromosomal coordinates as per the UCSC Genome Browser (http://genome.ucsc.edu/) using the sno/miRNA prediction tracks of the Mar.2006 (NCBI36/hg18) assembly.

### Prediction of miRNA target genes

To understand the function of CNV-miRNAs, we assessed validated and putative miRNA target genes in the context of two miRNA databases, mirDB (http://www.mirdb.org/)^28^ and miRWalk 3.0 (http://mirwalk.umm.uni-heidelberg.de/)^29^. The validated gene targets of miRNAs were obtained using the filtering function in the miRWalk 3.0 database. GeneCards (https://www.genecards.org/) and STRING v11 (https://string-db.org/)^30^ were used for the functional annotation of miRNA target genes. Additionally, the GTEx portal (https://gtexportal.org/home/) was used to examine the tissue-specific expression of the validated and/or putative miRNA target genes.

### Comparison of CNV-miRNAs in PEN-SCZ versus non-PEN-SCZ

First, the average length of total CNVs in PEN-SCZ versus non-PEN-SCZ groups was compared. Second, the average number of genes within CNVs was obtained. Third, the total number of miRNAs within CNVs was compared between the two groups. Forth, the number of patients harboring CNV-miRNAs was determined. Fifth, the average number of miRNAs per patient was compared. Finally, the number of patients with rates of miRNA genes per total genes within CNVs greater than 50% was compared between PEN-SCZ and non-PEN-SCZ groups.

### Gene ontology and pathway analyses

Gene ontology (GO) analysis was performed with all miRNAs observed within CNVs in each group, using MetaCore (Clarivate Analytics), a web-based licensed tool for comprehensive pathway analyses^31^. Gene set enrichment analysis of miRNA target genes was further performed to characterize the pathways affected in the PEN-SCZ group, also using MetaCore^31^.

### Statistical analysis

With respect to the demographic data, the average age, age of onset, plasma pentosidine levels, and the average of chlorpromazine equivalent doses were compared using the Student’s *t*-test (two-tailed). The rate of treatment resistant SCZ between PEN-SCZ and non-PEN-SCZ was evaluated by one-sided Fisher’s exact test. Regarding the CNV-miRNA analysis, the average length of total CNVs, number of genes within CNVs, and number of miRNAs per patient were assessed by Student’s *t*-test. To evaluate the differences in the rate of CNV-miRNAs between PEN-SCZ and non-PEN-SCZ groups, one-sided Fisher’s exact tests were used. If no variants were found in the two-by-two table, the OR was calculated after a 0 cell correction (0.5 was added to all cells), conducted to reduce bias^26^. Additionally, with respect to the in silico prediction of miRNA target genes using miRWalk, the *p* values were calculated from a random-forest-based approach using TarPmiR for miRNA target site prediction. A *p* value of <0.05 was considered significant.

### Power analysis

Power analysis was conducted using the Genetic Association Study (GAS) Power Calculator^27,32^. The following parameter assumptions were used to estimate power:^27^ prevalence of disease = 0.007 and α = 0.05 in the context of dominant model. Disease allele frequency of CNVs in the general population was obtained from Database for Genomic Variants (DGV) (http://dgv.tcag.ca/dgv/app/home). The genotype relative risk was calculated from the OR obtained using the Fisher’s exact test.

## Results

### CNV-miRNA genes are enriched in SCZ patients with enhanced glycation/oxidative stress

In this study, we compared the number of CNV-miRNAs between SCZ patients with high plasma pentosidine levels (PEN-SCZ) and those without the accumulation of plasma pentosidine (non-PEN-SCZ). Based on the power analysis, the expected power to detect significant CNV-miRNAs with an OR of 5.0 was 0.638 (Supplementary Table S1).

**Fig. 1:**
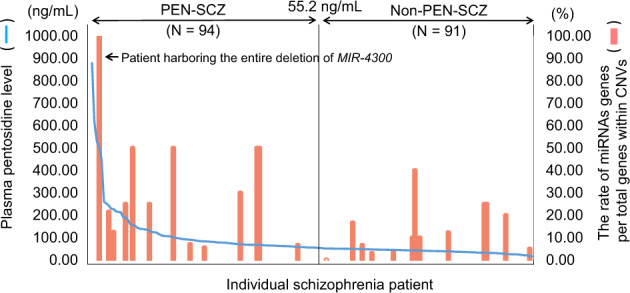
Plasma pentosidine levels and the rate of miRNA genes per total genes within CNVs in schizophrenia patients. In the genome-wide CNV-miRNA analysis, we compared the rate of miRNA genes per total genes within CNVs in PEN-SCZ versus non-PEN-SCZ. MiRNA-enriched CNVs, defined as a rate of miRNA genes in genes within CNVs >50%, was significantly higher in PEN-SCZ versus non-PEN-SCZ (*p* = 0.032).

Ninety-four patients (50.8%) with SCZ exhibited high plasma pentosidine levels, while 91 patients (49.2%) did not; these were classified as the PEN-SCZ and non-PEN-SCZ groups, respectively. We found a larger size of rare CNVs in the PEN-SCZ group, with 3.6 versus 0.4 Mb for PEN-SCZ and non-PEN-SCZ, respectively (Table 1 and Supplementary Fig. S1). Additionally, the number of genes within CNVs was 20.5-fold higher in PEN-SCZ versus non-PEN-SCZ (Table 1 and Supplementary Fig. S2). Regarding the number of miRNAs within CNVs, it was also 9.8-fold higher in PEN-SCZ versus non-PEN-SCZ, suggesting that CNV-miRNA genes were enriched in the context of PEN-SCZ (Table 1). On the other hand, although the number of miRNA genes within CNVs was larger in the PEN-SCZ group, the rate of patients harboring CNV-miRNAs was almost the same (*N* = 13). Next, we assessed the rate of miRNA genes in total genes within CNVs to adjust the effect of the CNV size; interestingly, miRNA-enriched CNVs, defined as per a miRNA-CNV rate >50%, were significantly higher in PEN-SCZ versus non-PEN-SCZ (Table 1 and Fig. 1, *p* = 0.032). Regarding the treatment resistant phenotype, clinical data from 98 SCZ subjects were available. Among them, 22 patients (39.3%) with PEN-SCZ were treatment resistant, and five patients (11.9%) with non-PEN-SCZ presented treatment resistant phenotype (*p* = 0.0022). A summary of the individual miRNAs within CNVs in PEN-SCZ and non-PEN-SCZ is presented in Supplementary Table S2. Of note, one miRNA, miR-4768, overlapped between the PEN-SCZ and non-PEN-SCZ groups, suggesting that it has little contribution to the difference in the plasma pentosidine levels. Therefore, this miRNA was omitted from further analyses.

### GO analysis suggests that the origin of the disruption in neurodevelopmental events is different in PEN-SCZ versus non-PEN-SCZ

A comparison of the GO properties of miRNAs within CNVs between PEN-SCZ and non-PEN-SCZ patients suggested that miRNAs involved in “midbrain dopamine neuron differentiation” (14.3%; 2.4 × 10^−7^) and “neuron–neuron synaptic transmission” (20.0%; 1.2 × 10^−7^) were likely to be affected in PEN-SCZ (Supplementary Table S3). On the other hand, “olfactory bulb interneuron differentiation,” was affected in non-PEN-SCZ (9.1%; 3.2 × 10^−4^), suggesting that different molecular mechanisms may be behind the etiology of the two SCZ subtypes.

### Notable miRNAs in patients with PEN-SCZ

Although the sample size and the number of observations were small, several notable CNV-miRNAs were identified in patients with PEN-SCZ with pentosidine plasma levels higher than 100 ng/mL in this study (Table 2). The main notable CNV-miRNAs are presented below.

**Table 2:** Copy number variable miRNAs in schizophrenia patients with accumulation of pentosidine.

Sample ID	Pentosidine (ng/mL)	Gender	Age	Disease-onset	Cytoband	CNV (del/dup)	microRNAs	Main target genes		
								GeneID	Annotation	Gene function
PEN-SCZ1	505.433	M	60	19	11q14.1	Del	MIR4300	*CACNA1C*	Voltage-dependent L-type calcium channel subunit alpha-1C	involved in calcium-dependent hormone or neurotransmitter release
								*HS6ST1*	Heparan-sulfate 6-O-sulfotransferase 1	critical for normal neuronal neuron branching and establishing neuronal connectivity
								*PYCR1*	Pyrroline-5-carboxylate reductase 1	involved in the cellular response to oxidative stress
								*DRD2*	Dopamine receptor D2	Dopamine receptor D2
								*MeCP2*	Methyl-CpG Binding Protein 2	plays an essential role in mammalian development including cognition
PEN-SCZ2	243.562	M	60	28	Xp22.33-p11.1Dup		MIR4767	*CPLX1*	Complexin 1, also known as synaphin 2	regulates a late step in exocytosis of synaptic vesicles when releasing neurotransmitters
PEN-SCZ3	189.341	F	60	34	Xp22.31	Dup				
PEN-SCZ4	227.38	M	32	23	10p15.3	Dup	MIR5699	*G6PC*	Glucose-6-phosphatase	a key enzyme in homeostatic regulation of blood glucose levels and glucose production
								*GRIN2B*	NMDA receptor subunit NR2B	plays a pivotal role in synaptic plasticity and cognition
								*MDGA1*	MAM domain containing glycosylphosphatidylinositol anchor 1	a binding partner of postsynaptic neuroligins, and involved in synapse development
PEN-SCZ5	156.819	F	52	25	19p13.11	Del	MIR640	*GSR*	*Glutathione reductase*	a central enzyme of cellular antioxidant defense
								*FXN*	*Frataxin, a mitochondrial protein*	plays a role in the protection against iron-catalyzed oxidative stress
								*ATCAY*	*Caytaxin*	involved in the postnatal maturation of the cerebellar cortex
PEN-SCZ6	131.812	M	52	23	8p23.1-p22	Del	MIR3926	*SHANK2*	*SH3 and multiple ankyrin repeat domains protein 2*	an adapter protein in the excitatory synapses that interconnects NMDA receptor and mGluRs, organizar of the dendritic spine
								*PCDHB14*	*Protocadherin Beta 14*	involved in establishing neuronal connectivity
								*PAX6*	*Paired Box 6*	involved in insulin signaling and brain development
PEN-SCZ7	100.931	M	40	20	18p11.21-q11.1	Dup	MIR3156	*TXNL1*	*Thioredoxin like 1*	a member of antioxidative thioredoxin system
								*SNAP29*	*Synaptosome associated protein 29*	mediate synaptic vesicle membrane docking and fusion to the plasma membrane

### CNV-miRNAs involved in the regulation of *CACNA1C* and *DRD2*

We identified one patient with PEN-SCZ presenting with extremely high plasma pentosidine levels (PEN = 505.4 ng/ml, Table 2 and Supplementary Fig. S3). This patient developed SCZ at the age of 19 and was considered a treatment-resistant patient. The patient carried a single CNV at 11q14.11 (OR = 2.94, 95% CI: 0.12–73.01), affecting only the miRNA-4300 (miR-4300) encoding gene, *MIR4300*. Interestingly, the in silico analysis identified 84 validated target genes of miR-4300 including *CACNA1C*, *HS6ST1*, and *PYCR1* (Table 2). *CACNA1C* encodes voltage-dependent L-type calcium channel subunit alpha-1C, which is involved in calcium-dependent hormone and neurotransmitter release. HS6ST1 encodes heparan-sulfate 6-*O*-sulfotransferase 1, with roles in neuron branching and neuronal connectivity, critical for normal neuronal development. *PYCR1* encodes pyrroline-5-carboxylate reductase 1, which catalyzes the last step in the biosynthesis of proline and is involved in the cellular response to oxidative stress. The putative target genes of miR-4300 are listed in Supplementary Table S4, including *DRD2* and *MECP2* that encode dopamine receptor D2 and methyl-CpG binding protein 2, respectively.

Notably, gene set enrichment analysis in the context of the validated target genes of miR-4300 suggested the enrichment of the following GO processes: (1) synaptic membrane adhesion to the extracellular matrix (27.3%; 5.5 × 10^−24^), (2) response to hormone (40.0%; 2.0 × 10^−8^), and (3) postsynaptic endosome to lysosome and postsynaptic neurotransmitter receptor diffusion (26.0%; 4.6 × 10^−34^) (Supplementary Fig. S4). Additionally, the putative target genes of miR-4300 were also linked to “serotonin receptor signaling”, one of the main targets of typical and atypical antipsychotic drugs.

### CNV-miRNA involved in glutamate signaling

Sixty-nine CNV-miRNAs were found in at least two patients with PEN-SCZ. In particular, CNVs in the miR-4767 gene, *MIR4767*, were shared in two patients with relatively high plasma pentosidine levels (PEN >100 ng/ml, Table 2). Of note, given the small number of observations of this CNV-miRNA due to the limited sample size, the OR was 4.95 (95%CI:0.23–104.45). Additionally, the in silico analysis revealed *CPLX1* (encoding complexin 1, also called synapsin 2 which regulates a late step in the exocytosis of synaptic vesicles) as a target gene of miR-4767 (Table 2). Interestingly, gene set enrichment analysis of the target genes of miRNA-4767 suggested that miRNA-4767 may be involved in the “positive regulation of glutamate secretion” (20.0%; 9.5 × 10^−11^) (Table 3).

**Table 3:** Gene set enrichment analysis of target genes for the copy number variable miRNAs in schizophrenia patients with accumulation of pentosidine.

Disrupted miRNAs	Pathways affected by target genes disrupted by CNV-miRNAs	
	Validated	Putative
miR-4300	Synaptic membrane adhesion to extracellular matrix (27.3%; 5.5 × 10^−24^)	G protein-coupled serotonin receptor signaling pathway (22.7%; 4.1 × 10^−19^)
	Response to hormone (40.0%; 2.0 × 10^−8^)	Serotonin receptor signaling (22.7%; 1.7 × 10^−18^)
	Postsynaptic endosome to lysosome, postsynaptic neurotransmitter receptor diffusion (26.0%; 4.6 × 10^−34^)	Cognition (40.9%; 3.1 × 10^−18^),
miR-4767	Negative regulation of establishment of endothelial barrier	Positive regulation of neuron death
	(13.0%; 8.7 × 10^−10^)	(36.7%; 5.6 × 10^−25^)
	Neuropeptide signaling pathway	Positive regulation of neuron apoptotic process
	(21.7%; 2.6 × 10^−7^)	(32.7%; 8.3 × 10^−25^)
	Positive regulation of glutamate secretion	
	(20.0%; 9.5 × 10^−11^)	
miR-5699	Synaptic membrane adhesion to extracellular matrix	Neuropeptide signaling pathway
	(21.7%; 1.0×10^−25^)	(32.0%; 1.6 × 10^−12^)
	Synaptic vesicle cytoskeletal transport	Circadian sleep/wake cycle process
	(17.4%; 2.7 × 10^−17^)	(20.0%; 7.9 × 10^−13^)
		G protein-coupled receptor signaling pathway
		(60.0%; 3.9 × 10^−10^)
miR-640	Negative regulation of insulin secretion response to glucose stimulus	GABAergic synaptic transmission
	(25.0%; 1.2 × 10^−12^)	(18.0%; 2.0 × 10^−14^)
	Long-term synaptic potentiation	Response to oxidative stress
	(25.0%; 6.2 × 10^−9^)	(33.3%; 1.2 × 10^−9^)
miR-3926	N/A*	Glutamate receptor signaling pathway
		(42.9%; 1.9 × 10^−12^)
		Wnt signaling pathway involved in midbrain dopaminergic neuron differentiation
		(17.4%; 7.5 × 10^−10^)
		Glucose metabolic process (23.5%; 6.5 × 10^−6^)
miR-3156-5p	Cellular response to phenylpropanoid (antioxidants) (100.0%; 2.6 × 10^−4^)	Positive regulation of neuron death (44.0%; 8.8 × 10^−17^)
	Cellular response to hydroxyisoflavone (antioxidants) (100.0%; 2.6 × 10^−4^)	GABA signaling pathway (56.0%; 1.1 × 10^−16^)
	Cellular response to genistein (antioxidants) (100.0%; 2.6 × 10^−4^)	
	Gamma-aminobutyric acid secretion (12.5%; 4.7 × 10^−13^)	

*Validated gene targets were not available.

### CNV-miRNAs are involved in glycation/oxidative stress and synaptic dysfunction

Additionally, CNVs in genes encoding miR-5699, miR-640, miR-3926, and miR-3156-5p may provide suggestive insights into the link between glycation/oxidative stress and synaptic dysfunction in the PEN-SCZ group (PEN >100 ng/mL) (Table 2). The notable targets of these miRNAs are presented in Table 2. MiR-5699 was disrupted by CNVs in a PEN-SCZ patient who exhibited 277.38 ng/ml plasma pentosidine (Table 2). The validated target genes of miR-5699 included *G6PC*, *GRIN2B*, and *MDGA1* (Table 2). *G6PC* encodes glucose-6-phosphatase, a key enzyme responsible for glucose production; *GRIN2B* encodes the NMDA receptor subunit NR2B that plays a pivotal role in synaptic plasticity and cognition; *MDGA1* encodes a MAM domain containing the glycosylphosphatidylinositol anchor 1, known as a negative regulator of synapse development and its expression is brain-specific (Supplementary Fig. S5). Of note, gene set enrichment analysis of the miR-5699 target genes implicated the possible involvement of the “synaptic membrane adhesion to the extracellular matrix,” “sleep,” and “regulation of neuronal death” processes (Table 3).

CNVs in the miR-640 gene (*MIR640*) were also found in one patient with PEN-SCZ (OR = 2.94, 95%CI: 0.12–73.01). Importantly, oxidative stress genes, including *GSR* and *FXN*, were included as its validated targets (Table 2)*. GSR* encodes glutathione reductase, a central enzyme of the cellular antioxidant defense. *FXN* encodes frataxin, a mitochondrial protein that plays a role in the protection of neurons against iron-catalyzed oxidative stress at puberty. Another validated target of miR-640 was *ATCAY*, which encodes caytaxin, involved in the postnatal maturation of the cerebellar cortex. Of note, based on the gene set enrichment analysis, the validated miR-640 target genes were enriched in the pathways involved in the “negative regulation of insulin secretion in response to glucose stimulus” (25.0%; 1.2 × 10^−12^) and “long-term synaptic potentiation (25.0%; 6.2 × 10^−9^),” suggesting the possible link between glycation stress and synaptic function (Table 3). Additionally, the putative miR-640 target genes were enriched in “GABAergic synaptic transmission” (18.0%; 2.0 × 10^−14^) and “response to oxidative stress” (33.3%; 1.2 × 10^−9^, Table 3).

MiR-3926 was also noted in one patient with PEN-SCZ (Table 2). Although no validated target genes were identified as per in silico analysis, predicted target genes included *SHANK2*, *PCDHB14*, and *PAX6*. *SHANK2* encodes SH3 and multiple ankyrin repeat domains protein 2, an adapter protein in the postsynaptic density of excitatory synapses that interconnects postsynaptic NMDA-type and metabotropic glutamate receptors (mGluRs); SHANK2 plays a pivotal role in the organization of the dendritic spine; *PCDHB14*, encoding protocadherin beta 14, plays a critical role in the establishment of neuronal connectivity; *PAX6* encodes paired box 6 and is involved in insulin signaling and brain development (Table 2). The expression of PAX6 is brain-specific (Supplementary Fig. S6). In line with these results, according to the gene set enrichment analysis, pathways related to synaptic function were enriched, such as the “glutamate receptor signaling pathway” (42.9%; 1.9 × 10^−12^, Table 3).

MiR-3156-5p, possibly involved in GABA signaling and oxidative stress was also observed in one patient with PEN-SCZ. The validated target genes of miR-3156-5p included *TXNL1*, encoding thioredoxin like 1, a member of the antioxidative thioredoxin system (Table 2). Gene set enrichment analysis on miR-3156-5p target genes suggested their involvement in GABA receptor signaling and oxidative stress, similar to the results obtained in the context of miR-640 (Table 3). The comprehensive list of the validated and putative miRNA target genes is provided in Supplementary Table S5.

## Discussion

In the present study, we examined the frequency of CNV-miRNAs in SCZ with and without accumulated plasma pentosidine, including the GO properties regulated by those miRNAs. Gene set enrichment analysis in the context of miRNA target genes found in rare CNVs was further performed for a better understanding of the molecular pathogenesis of PEN-SCZ. As a result, we observed that PEN-SCZ harbored 9.8-fold more miRNA-enriched CNVs than non-PEN-SCZ, possibly affecting different neuronal developmental events. In fact, analyses of individual CNV-miRNAs in PEN-SCZ suggested that miRNAs related to glycation/oxidative stress, brain maturation, and synaptic network, especially glutamate/GABA receptor signaling may be affected. Of note, although our sample size was limited, several notable CNV-miRNAs of interest were observed and suggestive evidence potentially linking glycation/oxidative stress and synaptic function via CNV-miRNAs was modestly disclosed.

Previously, it has been reported that more miRNAs are disrupted by rare CNVs in SCZ patients compared to controls^19^. For example, Warnica et al reported that patients with SCZ harbored 3.29-fold more CNV-miRNAs^19^. In this study, we found that 9.8-fold more miRNA genes were affected by rare CNVs in patients with PEN-SCZ versus non-PEN-SCZ, suggesting that the dysregulation of miRNAs may also be involved in the etiology of PEN-SCZ. Given that PEN-SCZ patients were reported to exhibit a clinical phenotype resistant to the current antipsychotic treatments^23,24^ and so as in our cohort (Table 1), we hypothesize this might be due to the high number of CNV-miRNA genes affecting multiple pathways. One fact potentially supporting this hypothesis is the deletion of *MIR4300*: the expression of *DRD2*, targeted by miR-4300, was possibly upregulated, making this patient resistant to the current antipsychotic drugs which mainly block DRD2.

Regarding the GO analysis, a previous CNV-miRNA study reported that neurodevelopmental pathways including axonogenesis and neuron projection development were affected in SCZ^19^. Our study, particularly the GO analysis, supports this notion that neuronal developmental events may be affected via CNV-miRNAs in SCZ. Interestingly, we found that the origins of the phenotype appeared to be different between PEN-SCZ and non-PEN-SCZ, which may also be behind the resistance to treatment with antipsychotic drugs in PEN-SCZ.

**Fig. 2:**
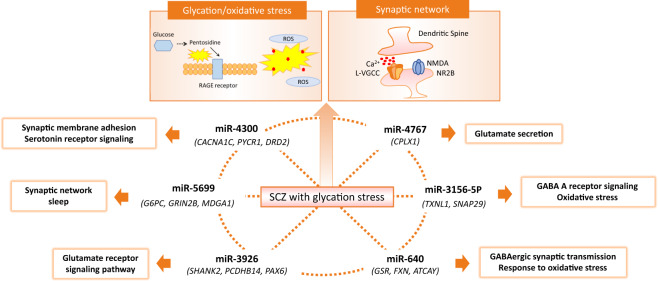
Diagram representing the possible link between glycation/oxidative stress and the synaptic function mediated by CNV-miRNAs. In this study, we identified several interesting copy number variable miRNAs (CNV-miRNAs) in the SCZ subtype characterized by accumulated plasma pentosidine. *MIR4300*, targeting recurrent SCZ risk genes such as *CACNA1C* and *DRD2* was entirely deleted in the patient with SCZ who showed extremely high plasma pentosidine. CNV-miRNAs, such as miR-5699, miR-3926, miR-640, and miR-3156-5p, associated with oxidative stress and synaptic function, including glutamate and GABA receptor signaling, were identified in other SCZ patients with high plasma pentosidine. Of note, two patients with PEN-SCZ shared the CNV-miRNA, miR-4767, possibly involved in glutamate secretion as per the gene set enrichment analysis. Our results may indicate that the miRNAs involved in glycation, oxidative stress, and synaptic function are disrupted by CNVs in SCZ patients with the enhanced glycation and oxidative stress subtype.

Regarding the notable CNV-miRNAs identified in this study, *MIR4300* has been reported as a risk gene for adolescent idiopathic scoliosis (AIS)^33^ as well as for SCZ in the latest large-scale GWAS^7^. Here, we identified the deletion of the entire *MIR4300* in a patient with SCZ showing extremely high plasma pentosidine. It is interesting to note that the onset during adolescence is a shared feature between AIS and SCZ. *MIR4300* is highly expressed in the testis and brain and thought to bind to the hormone regulating genes associated with the disease-onset during adolescence^33^. Our gene set enrichment analysis results for the miR-4300 target genes supported this notion, showing its involvement in hormonal regulation. In fact, of the 14 novel genome-wide significant loci identified in the large-scale GWAS on AIS, using 79,211 Japanese individuals, four genes overlapped with SCZ risk genes, namely *CSMD1*, *PLXNA2*, *MTMR11*, and *CDK13*^7,8,34^. In addition, the target genes of miR-4300 included *CACNA1C*, *DRD2*, *MECP2*, and *PYCR1*, the most recurrent SCZ risk genes^7,8,35^. Under normal conditions, miR-4300 binds to the 3′UTR of *CACNA1C* and negatively regulates its expression, resulting in protection against oxidative stress^36^. Although in this study we identified only one case, we still speculate that SCZ patients harboring the entire deletion of *MIR4300* lack this protective mechanism and are vulnerable to oxidative stress. *PYCR1*, another miR-4300 target gene that is linked to glycation stress^37^, interacts with DJ-1, one of the deglycation critical enzymes, and prevents neuronal damage caused by the accumulation of advanced glycation end product (AGE) like pentosidine, the marker of glycation^37,38^. Therefore, the deletion of *MIR4300* might be involved in both enhanced glycation stress and oxidative stress through *PYCR1* and *CACNA1C* (Fig. 2).

Moreover, we identified G6PC, a key glucose metabolism enzyme, and GSR, the main enzyme of the antioxidative stress system, as the validated miRNA target genes in SCZ patients with accumulated plasma pentosidine. Pentosidine is continuously formed; however, this occurs more rapidly under excess oxidative stress and hyperglycemia, making it a biomarker of both glycation and oxidative damage to proteins^39^ In this context, it is particularly interesting that enzymes involved in glucose metabolism and oxidative stress were both targeted by the CNV-miRNAs in SCZ patients with high plasma pentosidine.

In accordance with the NMDA receptor hypofunction hypothesis^40,41^, *GRIN2B*, encoding the NMDA receptor subunit NR2B, was identified as a validated target gene of miR-5699, affected in patients with PEN-SCZ. Additionally, in this study, another patient with high plasma pentosidine harbored the deletion of the entire *MIR-3926* that targets *SHANK2*, an adapter protein that interconnects postsynaptic NMDA receptor and mGluR5^42^. We might, therefore, speculate that the shared miRNA target genes are related to NMDA receptor signaling molecules, while the other target genes may contribute to generate the heterogeneous phenotypes observed in SCZ.

Interestingly, in the context of miR-640 and miR-3156-5p, similar pathways were enriched as per the miRNA target genes, such as GABAergic synaptic neurotransmission and oxidative stress (Table 3). Methylglyoxal, a key precursor of AGEs such as pentosidine^43^, acts as a GABAA receptor partial agonist^44^. Furthermore, previous studies reported that the GABAergic neurons are more vulnerable to oxidative stress^45^, raising the possibility that they might be also vulnerable to glycation stress. The high energy demand for the presynaptic release of GABA was previously discussed in brain diseases^46^. Our results may suggest that miRNAs targeting the GABAA receptor signaling pathway are disrupted by CNVs in a portion of SCZ patients with high plasma levels of pentosidine. Figure 2 illustrates a possible link between glycation/oxidative stress and the synaptic function mediated by CNV-miRNAs.

This study has several limitations. First, the sample size was small and the study was underpowered (Supplementary Table S1) as we analyzed CNV-miRNAs using patients with SCZ harboring rare CNVs. Although the clinical evaluation of the relationship between genomic factors and phenotypes in individual patients is needed to generate more data, a larger sample size is required in order not to miss other important CNV-miRNAs. Of note, evaluating the effect size of CNVs that are too rare to study in individual association studies has been in deep argument^47^. Of note, GO and gene set enrichment analyses may help to understand the pathway affected by the rare CNV-miRNAs, the reason why we performed them in this study. Second, our analyses included not only validated target genes for the miRNAs but also putative genes. The need for the prediction of miRNA target genes using an in silico computational approach is another limitation of the present study. Third, the cause of the increase in plasma pentosidine may be polygenic in nature and attributed to the sum of small individual effects. Although miR-4300 was identified as only one gene affected by CNVs, other genes in CNVs not coding for miRNAs coexisted suggesting that the etiology of SCZ is associated with a combined effect of CNVs on miRNAs and other genes. Forth, the possibility that other genetic and epigenetic factors such as methylation was not excluded.

In conclusion, although this is a preliminary study with a limited sample size, our integrative genome-wide CNV-miRNA analysis is the first to provide suggestive evidence that miRNA-enriched CNVs are more prevalent in PEN-SCZ versus non-PEN-SCZ, disrupting different neuronal developmental events. Of note, the analysis of individual CNV-miRNAs observed in PEN-SCZ implicated a possible regulatory link between glycation/oxidative stress and synaptic dysfunction in SCZ patients with enhanced glycation/oxidative stress. Therefore, targeting miRNAs within CNVs to restore the aberrant synaptic function may be a promising strategy for the development of personalized treatments for SCZ patients with enhanced glycation/oxidative stress.

## Supplementary information

Supplemental Material
