# Supplementary material for: Dysregulation of post-transcriptional modification by copy number variable microRNAs in schizophrenia with enhanced glycation stress
Source: Transl Psychiatry. 2021 May 28;11:331. doi: 10.1038/s41398-021-01460-1 (PMC8163801; doi:10.1038/s41398-021-01460-1)
Supplement: Supplementary file 1 — Supplemental Material [file 41398_2021_1460_MOESM1_ESM.docx]

**Supplementary Information for**

**Dysregulation of Post-transcriptional Modification by Copy Number Variable MicroRNAs in Schizophrenia with Enhanced Glycation Stress**

Akane Yoshikawa MD, PhD, Itaru Kushima MD, PhD, Mitsuhiro Miyashita MD, PhD, Kazuya Toriumi PhD, Kazuhiro Suzuki MD, Yasue Horiuchi PhD, Hideya Kawaji PhD, Shunya Takizawa MD, PhD, Norio Ozaki MD, PhD, Masanari Itokawa MD, PhD, Makoto Arai PhD*

***Corresponding Author:**

Makoto Arai PhD, Project Leader,

Schizophrenia Research Project, Department of Psychiatry and Behavioral Sciences, Tokyo Metropolitan Institute of Medical Science

2-1-6, Kamikitazawa, Setagaya-ku, Tokyo, 156-8506, Japan.

Tel: +81-3-6834-2380 Direct: 3824; Fax: +81-3-6834-2389

Email: arai-mk@igakuken.or.jp

**This file includes:**

- Supplementary Figures S1 to S6
- Supplementary Tables S1 to S5

**Supplementary Figures**

*
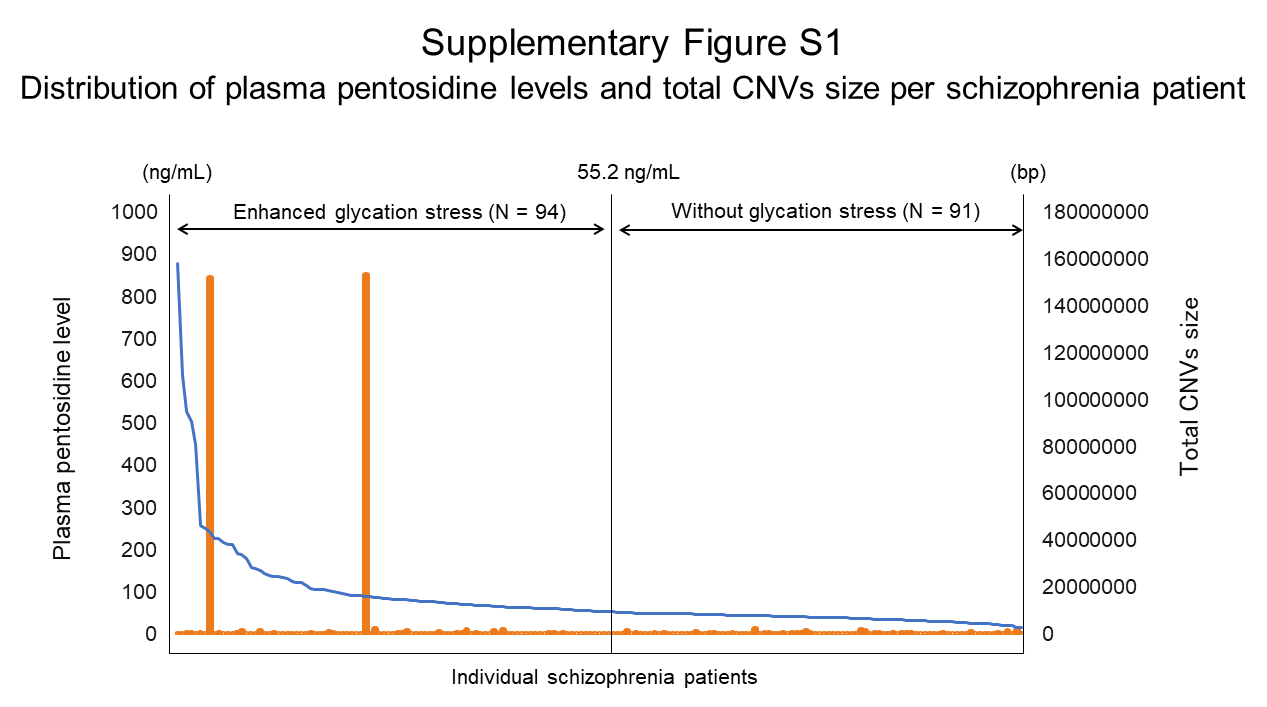
*

***Supplementary Figure S1***

***Distribution of the plasma pentosidine levels and total CNVs size per schizophrenia patient***

The average length of CNVs was 3.6 Mb in the PEN-SCZ group and 0.4Mb in the non-PEN-SCZ group, suggesting that larger deletions or duplications tend to be observed in the context of PEN-SCZ.

***
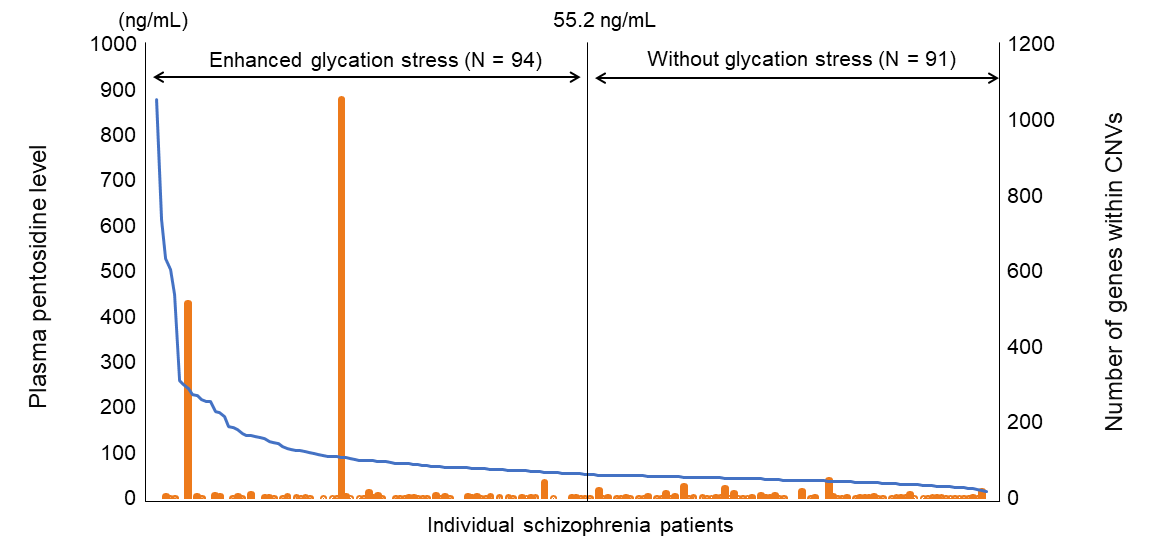
***

***Supplementary Figure S2***

***Distribution of the plasma pentosidine levels and total number of genes within CNVs per schizophrenia patient***

The average number of genes within CNVs was higher in PEN-SCZ, the enhanced glycation/oxidative stress subtype, than that in non-PEN-SCZ.

***
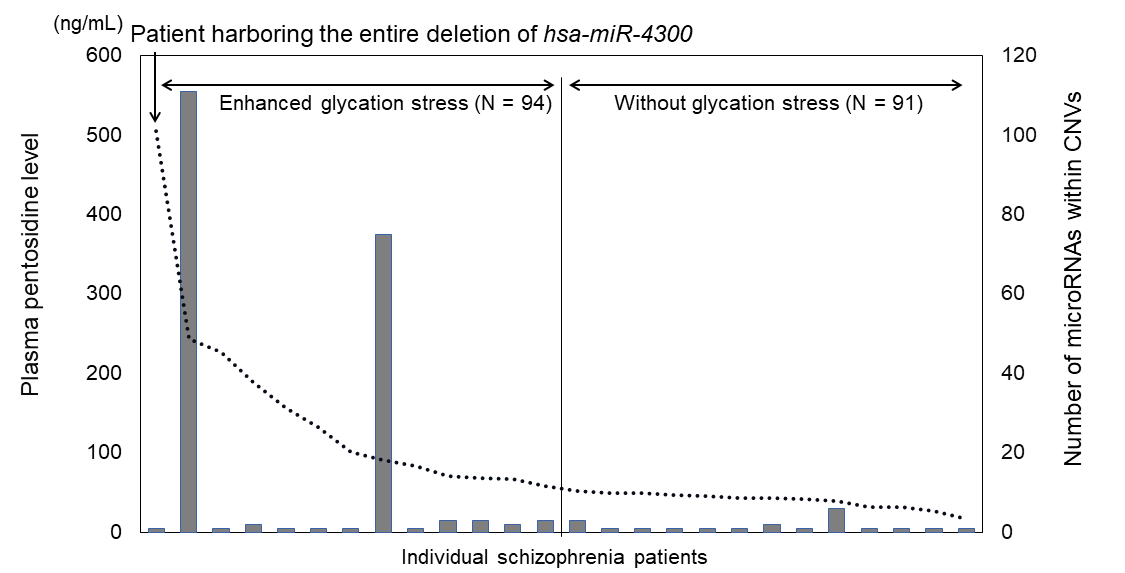
***

***Supplementary Figure S3***

***Distribution of the plasma pentosidine levels and total number of copy number variable miRNA genes per schizophrenia patient***

In schizophrenia patients with enhanced glycation and oxidative stress, the total number of copy number variable miRNA genes was enriched compared with those in the non-glycation/oxidative stress subtype.

***
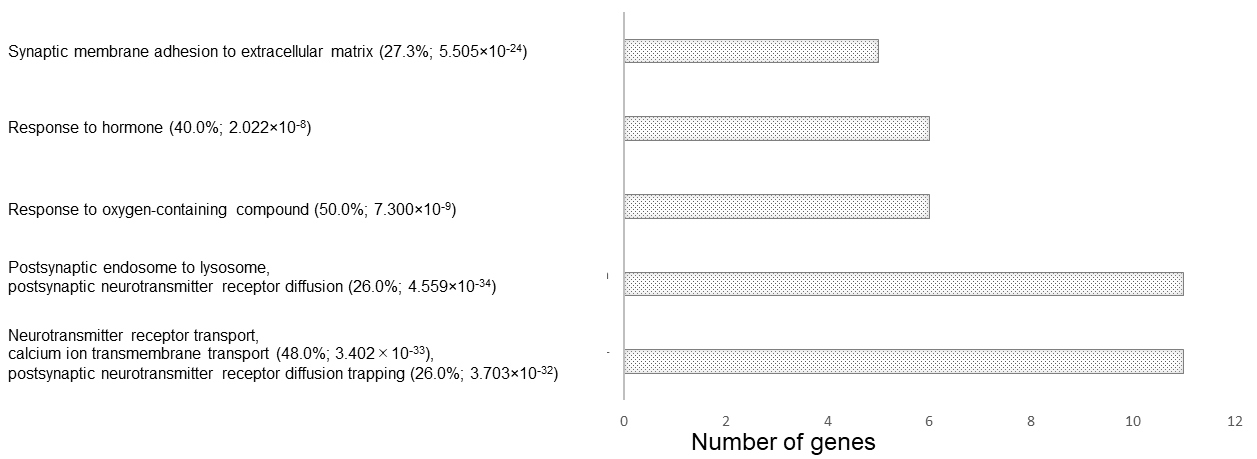
***

***Supplementary Figure S4***

***Gene set enrichment analysis of the validated gene targets of miR-4300***

*The gene set enrichment analysis using the validated targets of miR-4300 revealed enrichment of “synaptic membrane adhesion to the extracellular matrix” (27.3%; 5.5 × 10^-24^), “response to hormone” (40.0%; 2.0 × 10^-8^), and “postsynaptic endosome to lysosome and postsynaptic neurotransmitter receptor diffusion” (26.0%; 4.6 × 10^-34^).*

*
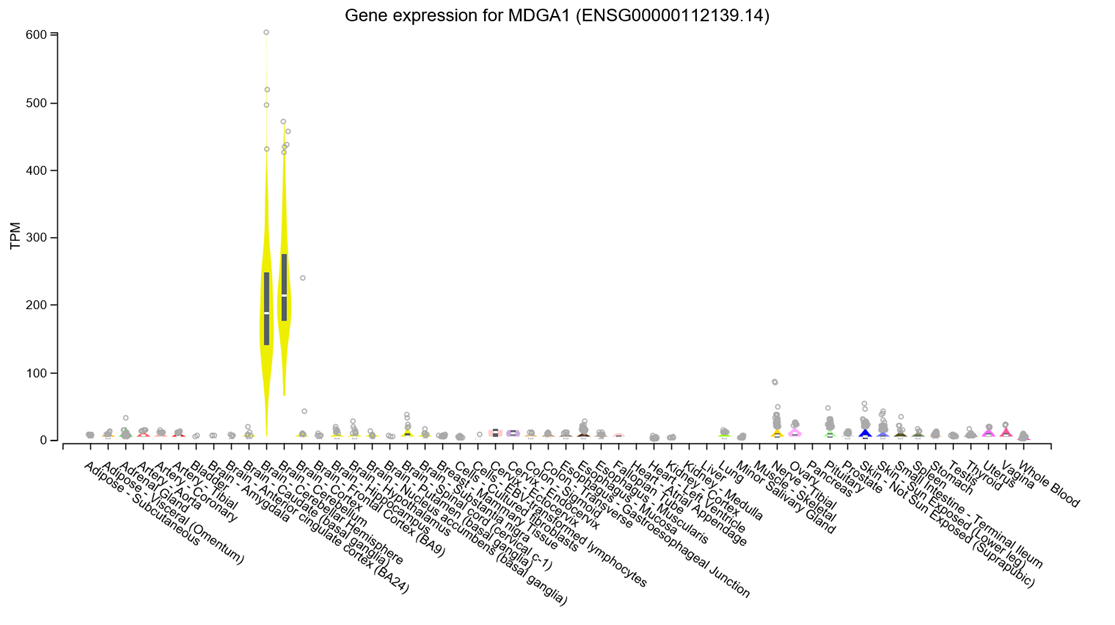
*

***Supplementary Figure S5***

***Brain-specific expression of the MDGA1***

According to the GTEx portal, the expression of *MDGA1*, the main target gene for miR-5699, is brain-specific.

***
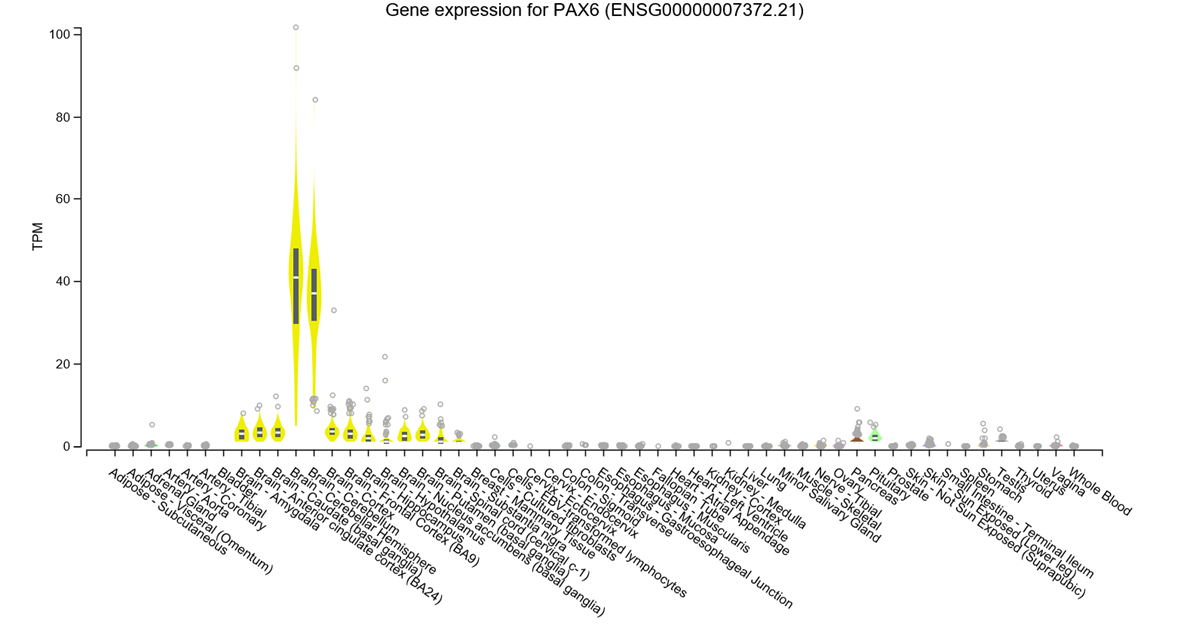
Supplementary Figure S6***

***Brain-specific expression of the PAX6***

According to the GTEx portal, the expression of *PAX6*, the main target gene for miR-3926, is brain-specific.

**Supplementary Tables**

***Supplementary Table S1***

***Power to detect associations with copy number variable microRNAs observed in patients with PEN-SCZ***

|  |  |  |  |  |  |  |
| --- | --- | --- | --- | --- | --- | --- |
| Cytoband | CNV (del/dup) | MicroRNAs | Disease allele frequency | Genotype relative risk | Power |  |
|  |  |  |  |  |  |  |
| 11q14.1 | Del | MIR4300 | 0.0130 | 2.94 | 0.332 |  |
| Xp22.31 | Dup | MIR4767 | 0.0001 | 4.95 | 0.056 |  |
| 10p15.3 | Dup | MIR5699 | 0.0001 | 2.94 | 0.052 |  |
| 19p13.11 | Del | MIR640 | 0.0001 | 2.94 | 0.052 |  |
| 8p23.1-p22 | Del | MIR3926 | 0.3040 | 2.94 | 0.852 |  |
| 18p11.21-q11.1 | Dup | MIR3156 | 0.0001 | 2.94 | 0.052 |  |

The power of this study (94 PEN-SCZ cases and 91 non-PEN-SCZ cases) to detect significant associations with the 6 copy number variable microRNAs is presented.

***Supplementary Table S2***

***Summary of miRNAs within CNVs in the context of PEN-SCZ and non-PEN-SCZ***

|  |  |  |  |  |  |  |  |
| --- | --- | --- | --- | --- | --- | --- | --- |
| Disease phenotype | Pentosidine (ng/mL) | PEN | Gender | Age | Cytoband | Number of miRNAs within CNVs | miRNAs |
| PEN-SCZ | 505.43 | High | male | 60 | 11q14.1 | 1 | MIR4300 |
| PEN-SCZ | 243.56 | High | male | 60 | Xp22.33-p11.1 | 111 | MIR1587,MIR188,MIR221,MIR222,MIR222,MIR23C,MIR362,MIR3690,MIR3915,MIR3937,MIR4666B,MIR4767,MIR4769,MIR4770,MIR500A,MIR500B,MIR501,MIR502,MIR532,MIR548AJ2,MIR548AM,MIR548AX,MIR548F5,MIR6089,MIR6134,MIR651,MIR660,MIR6857,MIR6894,MIR6895,MIR8088,MIR98,MIR1587,MIR4536-1 |
|  |  |  |  |  | Xq11.1-q28 |  | MIR105-1,MIR105-2,MIR106A,MIR1184-1,MIR1184-2,MIR1184-3,MIR1264,MIR1277,MIR1298,MIR1468,MIR18B,MIR1911,MIR1912,MIR19B2,MIR20B,MIR2114,MIR223,MIR224,MIR3202-1,MIR320D2,MIR325,MIR361,MIR363,MIR3672,MIR374A,MIR374B,MIR3978,MIR421,MIR424,MIR4329,MIR4330,MIR448,MIR450A1,MIR450A2,MIR450B,MIR452,MIR503,MIR503HG,MIR504,MIR505,MIR506,MIR507,MIR508,MIR509-1,MIR509-2,MIR509-3,MIR510,MIR513A1,MIR513A2,MIR513B,MIR513C,MIR514A1,MIR514A2,MIR514A3,MIR514B,MIR542,MIR545,MIR548AN,MIR548I4,MIR548M,MIR6087,MIR652,MIR676,MIR6858,MIR718,MIR764,MIR766,MIR767,MIR888,MIR890,MIR891A,MIR891B,MIR892A,MIR892B,MIR892C,MIR92A2,MIR934, |
| PEN-SCZ | 227.38 | High | male | 32 | 10p15.3 | 1 | MIR5699 |
| PEN-SCZ | 189.34 | High | female | 60 | Xp22.31 | 2 | MIR4767, MIR651 |
| PEN-SCZ | 156.82 | High | female | 52 | 19p13.11 | 1 | MIR640 |
| PEN-SCZ | 131.81 | High | male | 52 | 8p23.1-p22 | 1 | MIR3926-1 |
| PEN-SCZ | 100.93 | High | male | 40 | 18p11.21-q11.1 | 1 | MIR3156-2 |
| PEN-SCZ | 90.78 | High | male | 39 | 16p13.11 | 75 | MIR3179-2,MIR3180-2,MIR3670-2,MIR484,MIR6506,MIR6511A2,MIR6770-2,MIR-3180-1 |
|  |  |  |  |  | Xq21.31-q28 |  | MIR105-1,MIR105-2,MIR106A,MIR1184-1,MIR1184-2,MIR1184-3,MIR1264,MIR1277,MIR1298,MIR18B,MIR1911,MIR1912,MIR19B2,MIR20B,MIR2114,MIR224,MIR3202-1,MIR320D2,MIR363,MIR3672,MIR3978,MIR424,MIR4329,MIR4330,MIR448,MIR450A1,MIR450A2,MIR450B,MIR452,MIR503,MIR503HG,MIR504,MIR505,MIR506,MIR507,MIR508,MIR509-1,MIR509-2,MIR509-3,MIR510,MIR513A1,MIR513A2,MIR513B,MIR513C,MIR514A1,MIR514A2,MIR514A3,MIR514B,MIR542,MIR548AN,MIR548M,MIR6087,MIR652,MIR6858,MIR718,MIR764,MIR766,MIR767,MIR888,MIR890,MIR891A,MIR891B,MIR892A,MIR892B,MIR892C,MIR92A2,MIR934, |
| PEN-SCZ | 83.24 | High | female | 66 | 1q21.1 | 1 | MIR6736 |
| PEN-SCZ | 70.73 | High | male | 45 | 9p22.1-p21.3 | 3 | MIR4473,MIR4474,MIR491 |
| PEN-SCZ | 67.61 | High | male | 41 | 2q13 | 3 | MIR4267,MIR4436B1,MIR4436B2 |
| PEN-SCZ | 67.18 | High | male | 27 | 22q12.3 | 2 | MIR3909,MIR6069 |
| PEN-SCZ | 58.38 | High | male | 61 | 14q32.33 | 3 | MIR4507, MIR4537,MIR4539 |
| Non PEN-SCZ | 51.26 | Norm | female | 41 | 12p13.31 | 3 | MIR141,MIR200C |
|  |  |  |  |  | 22q12.3 |  | MIR4764 |
| Non PEN-SCZ | 49.32 | Norm | female | 58 | 17q25.1 | 1 | MIR3615 |
| Non PEN-SCZ | 48.67 | Norm | female | 75 | 11p11.2 | 1 | MIR3161 |
| Non PEN-SCZ | 47.22 | Norm | female | 78 | 1q42.13 | 1 | MIR4666A |
| Non PEN-SCZ | 44.77 | Norm | female | 50 | 1q21.1 | 1 | MIR6077 |
| Non PEN-SCZ | 43.10 | Norm | female | 63 | 22q12.1 | 1 | MIR5739 |
| Non PEN-SCZ | 42.61 | Norm | male | 48 | 7q33 | 2 | MIR490 |
|  |  |  |  |  | 8p23.3 |  | MIR596 |
| Non PEN-SCZ | 41.94 | Norm | male | 36 | 17p12-p11.2 | 1 | MIR1288 |
| Non PEN-SCZ | 38.65 | Norm | female | 33 | 22q11.21 | 6 | MIR1286,MIR1306,MIR185,MIR3618,MIR4761,MIR6816 |
| Non PEN-SCZ | 32.15 | Norm | male | 27 | 4q35.1 | 1 | MIR4455 |
| Non PEN-SCZ | 31.82 | Norm | male | 49 | 5q35.3 | 1 | MIR8089 |
| Non PEN-SCZ | 26.84 | Norm | female | 42 | 1p22.3 | 1 | MIR7856 |
| Non PEN-SCZ | 16.86 | Norm | female | 74 | 1q21.1 | 1 | MIR6077 |

Summary of the individual miRNAs within CNVs in PEN-SCZ and non-PEN-SCZ is presented.

***Supplementary Table S3***

***Gene ontology in the context of CNV-miRNAs in schizophrenia patients with and without the accumulation of pentosidine***

|  | |
| --- | --- |
|  |  |
| Gene Ontology | |
| PEN-SCZ ^a)^ | Non PEN-SCZ ^b)^ |
| midbrain dopamine neuron differentiation | olfactory bulb interneuron differentiation |
| (14.3%; 2.367×10^-7^) | (9.1%; 3.247×10^-4^) |
| neuron-neuron synaptic transmission |  |
| (20.0%; 1.247×10^-7^) |  |
| ^a^ schizophrenia with accumulated plasma pentosidine | |
| ^b^ schizophrenia without accumulated plasma pentosidine | |

A comparison of the GO properties of miRNAs within CNVs between PEN-SCZ and non-PEN-SCZ patients suggested that miRNAs involved in “midbrain dopamine neuron differentiation” (14.3%; 2.4 × 10^-7^) and “neuron–neuron synaptic transmission” (20.0%; 1.2 × 10^-7^) were likely to be affected in PEN-SCZ.

***Supplementary Table S4***

***Putative gene targets of miR-4300 disrupted in a schizophrenia patient with extremely high plasma pentosidine***

|  |  |  |  |  |
| --- | --- | --- | --- | --- |
| Target Rank | Target Score | miRNA | Gene Symbol | Gene Description |
| 1 | 96 | miR-4300 | *ATPAF1* | ATP synthase mitochondrial F1 complex assembly factor 1 |
| 2 | 96 | miR-4300 | *TNRC6B* | trinucleotide repeat containing 6B |
| 3 | 95 | miR-4300 | *PLXNA4* | plexin A4 |
| 4 | 93 | miR-4300 | *NOA1* | nitric oxide associated 1 |
| 5 | 93 | miR-4300 | *SMCR8* | Smith-Magenis syndrome chromosome region, candidate 8 |
| 6 | 92 | miR-4300 | *GINS2* | GINS complex subunit 2 |
| 7 | 92 | miR-4300 | *RASGRP3* | RAS guanyl releasing protein 3 |
| 8 | 92 | miR-4300 | *PRX* | periaxin |
| 9 | 92 | miR-4300 | *LY6G6C* | lymphocyte antigen 6 family member G6C |
| 10 | 92 | miR-4300 | *NUCB2* | nucleobindin 2 |
| 11 | 91 | miR-4300 | *MYCT1* | MYC target 1 |
| 12 | 91 | miR-4300 | *HIC1* | HIC ZBTB transcriptional repressor 1 |
| 13 | 90 | miR-4300 | *EDA2R* | ectodysplasin A2 receptor |
| 14 | 90 | miR-4300 | *DUSP23* | dual specificity phosphatase 23 |
| 15 | 90 | miR-4300 | *FBXO10* | F-box protein 10 |
| 16 | 90 | miR-4300 | *CLEC4E* | C-type lectin domain family 4 member E |

The putative target genes of miR-4300 are listed.

***Supplementary Table S5***

***Comprehensive list of the validated and putative miRNA target genes***

| Gene Symbol | miR^a^ | type | Target Score |
| --- | --- | --- | --- |
| EMX2 | miR-5699 | Predicted | >90 |
| CLMN | miR-5699 | Predicted | >90 |
| ACKR2 | miR-5699 | Predicted | >90 |
| WDHD1 | miR-5699 | Predicted | >90 |
| CLN8 | miR-5699 | Predicted | >90 |
| RTP1 | miR-5699 | Predicted | >90 |
| ZNF26 | miR-5699 | Predicted | >90 |
| PTPN7 | miR-5699 | Predicted | >90 |
| TNRC6C | miR-5699 | Predicted | >90 |
| MLPH | miR-5699 | Predicted | >90 |
| TBC1D7 | miR-5699 | Predicted | >90 |
| CCDC141 | miR-5699 | Predicted | >90 |
| EPO | miR-5699 | Predicted | >90 |
| MAP2K4 | miR-5699 | Predicted | >90 |
| DMRTC1B | miR-5699 | Predicted | >90 |
| DTD2 | miR-5699 | Predicted | >90 |
| DMRTC1 | miR-5699 | Predicted | >90 |
| GALNT13 | miR-5699 | Predicted | >90 |
| TLN1 | miR-4300 | Validated | NA |
| BICD2 | miR-4300 | Validated | NA |
| HAUS3 | miR-4300 | Validated | NA |
| PTPA | miR-4300 | Validated | NA |
| ABL1 | miR-4300 | Validated | NA |
| FIBCD1 | miR-4300 | Validated | NA |
| VAV2 | miR-4300 | Validated | NA |
| APOM | miR-4300 | Validated | NA |
| MAP3K2 | miR-4300 | Validated | NA |
| HS6ST1 | miR-4300 | Validated | NA |
| FOLR1 | miR-4300 | Validated | NA |
| PYCR1 | miR-4300 | Validated | NA |
| TNS4 | miR-4300 | Validated | NA |
| EFTUD2 | miR-4300 | Validated | NA |
| RPL22 | miR-4300 | Validated | NA |
| MINOS1-NBL1 | miR-4300 | Validated | NA |
| NBL1 | miR-4300 | Validated | NA |
| IFNLR1 | miR-4300 | Validated | NA |
| EXTL3 | miR-4300 | Validated | NA |
| PAQR5 | miR-4300 | Validated | NA |
| MSN | miR-4300 | Validated | NA |
| ERC1 | miR-4300 | Validated | NA |
| CACNA1C | miR-4300 | Validated | NA |
| GPRC5B | miR-4300 | Validated | NA |
| C16orf58 | miR-4300 | Validated | NA |
| ADGRG1 | miR-4300 | Validated | NA |
| PARD6B | miR-4300 | Validated | NA |
| TPD52L2 | miR-4300 | Validated | NA |
| SCD | miR-4300 | Validated | NA |
| MBOAT2 | miR-4300 | Validated | NA |
| E2F6 | miR-4300 | Validated | NA |
| AP1G1 | miR-4300 | Validated | NA |
| GLG1 | miR-4300 | Validated | NA |
| ZNF469 | miR-4300 | Validated | NA |
| DBNDD1 | miR-4300 | Validated | NA |
| ANKFY1 | miR-4300 | Validated | NA |
| ANKRD52 | miR-4300 | Validated | NA |
| NPTX2 | miR-4300 | Validated | NA |
| TBL1XR1 | miR-4300 | Validated | NA |
| CBX6 | miR-4300 | Validated | NA |
| CHCHD10 | miR-4300 | Validated | NA |
| TCN2 | miR-4300 | Validated | NA |
| IL2RB | miR-4300 | Validated | NA |
| ELFN2 | miR-4300 | Validated | NA |
| ZFAND4 | miR-4300 | Validated | NA |
| NEGR1 | miR-4300 | Validated | NA |
| KMT2D | miR-4300 | Validated | NA |
| LAD1 | miR-4300 | Validated | NA |
| ARL8A | miR-4300 | Validated | NA |
| NFASC | miR-4300 | Validated | NA |
| NUCKS1 | miR-4300 | Validated | NA |
| MAPKAPK2 | miR-4300 | Validated | NA |
| HOXA7 | miR-4300 | Validated | NA |
| AVL9 | miR-4300 | Validated | NA |
| ZNF662 | miR-4300 | Validated | NA |
| LIMD1 | miR-4300 | Validated | NA |
| NCKIPSD | miR-4300 | Validated | NA |
| UBE2D3 | miR-4300 | Validated | NA |
| PPIL6 | miR-4300 | Validated | NA |
| HMGN2 | miR-4300 | Validated | NA |
| WASF2 | miR-4300 | Validated | NA |
| HIVEP3 | miR-4300 | Validated | NA |
| SETD1B | miR-4300 | Validated | NA |
| ASXL2 | miR-4300 | Validated | NA |
| MCFD2 | miR-4300 | Validated | NA |
| NACC1 | miR-4300 | Validated | NA |
| CHERP | miR-4300 | Validated | NA |
| TTC9C | miR-4300 | Validated | NA |
| RELA | miR-4300 | Validated | NA |
| SPRY4 | miR-4300 | Validated | NA |
| IL5RA | miR-4300 | Validated | NA |
| LHFPL4 | miR-4300 | Validated | NA |
| NUP98 | miR-4300 | Validated | NA |
| SELENOS | miR-4300 | Validated | NA |
| PKD1 | miR-4300 | Validated | NA |
| MAT1A | miR-4300 | Validated | NA |
| CAMTA2 | miR-4300 | Validated | NA |
| SMCR8 | miR-4300 | Validated | NA |
| SLC47A1 | miR-4300 | Validated | NA |
| RASL10B | miR-4300 | Validated | NA |
| HMGA1 | miR-4300 | Validated | NA |
| KCNK5 | miR-4300 | Validated | NA |
| HSP90AB1 | miR-4300 | Validated | NA |
| PKHD1 | miR-4300 | Validated | NA |
| MB21D2 | miR-3926 | Predicted | NA |
| PIGA | miR-3926 | Predicted | NA |
| HOMER1 | miR-3926 | Predicted | NA |
| YIPF4 | miR-3926 | Predicted | NA |
| PTMA | miR-3926 | Predicted | NA |
| AREL1 | miR-3926 | Predicted | >90 |
| LSM12 | miR-3926 | Predicted | >90 |
| SHANK2 | miR-3926 | Predicted | >90 |
| KCNG3 | miR-3926 | Predicted | >90 |
| ABLIM3 | miR-3926 | Predicted | >90 |
| RYK | miR-3926 | Predicted | >90 |
| KIAA1549 | miR-3926 | Predicted | >90 |
| DHX15 | miR-3926 | Predicted | >90 |
| ZNF318 | miR-3926 | Predicted | >90 |
| ANKFY1 | miR-3926 | Predicted | >90 |
| ATP8B2 | miR-3926 | Predicted | >90 |
| SP1 | miR-3926 | Predicted | >90 |
| PTPRB | miR-3926 | Predicted | >90 |
| SMAD7 | miR-3926 | Predicted | >90 |
| ATP6V1D | miR-3926 | Predicted | >90 |
| TNFAIP1 | miR-3926 | Predicted | >90 |
| VDAC3 | miR-3926 | Predicted | >90 |
| BIRC6 | miR-3926 | Predicted | >90 |
| PRR15L | miR-3926 | Predicted | >90 |
| SCG5 | miR-3926 | Predicted | >90 |
| LOXL1 | miR-3926 | Predicted | >90 |
| PRRC2B | miR-3926 | Predicted | >90 |
| TES | miR-3926 | Predicted | >90 |
| EIF4EBP2 | miR-3926 | Predicted | >90 |
| ADGRD1 | miR-3926 | Predicted | >90 |
| JAM3 | miR-3926 | Predicted | >90 |
| PAX6 | miR-3926 | Predicted | >90 |
| NAA15 | miR-3926 | Predicted | >90 |
| CYSTM1 | miR-3926 | Predicted | >90 |
| FOXG1 | miR-3926 | Predicted | >90 |
| SELENOT | miR-3926 | Predicted | >90 |
| RBL1 | miR-3926 | Predicted | >90 |
| PLCB1 | miR-3926 | Predicted | >90 |
| ATXN3 | miR-3926 | Predicted | >90 |
| SBSPON | miR-3926 | Predicted | >90 |
| SLC30A5 | miR-3926 | Predicted | >90 |
| KDM8 | miR-3926 | Predicted | >90 |
| RBPJ | miR-3926 | Predicted | >90 |
| SCRT2 | miR-3926 | Predicted | >90 |
| VPS26A | miR-3926 | Predicted | >90 |
| MMP24 | miR-3926 | Predicted | >90 |
| TULP4 | miR-3926 | Predicted | >90 |
| JRKL | miR-3926 | Predicted | >90 |
| ACPP | miR-3926 | Predicted | >90 |
| PCDHB14 | miR-3926 | Predicted | >90 |
| POLR1B | miR-3926 | Predicted | >90 |
| ESYT2 | miR-3926 | Predicted | >90 |
| MUC15 | miR-3926 | Predicted | >90 |
| ATP6V0E1 | miR-3926 | Predicted | >90 |
| BRAP | miR-3926 | Predicted | >90 |
| CDH11 | miR-3926 | Predicted | >90 |
| KLF6 | miR-3926 | Predicted | >90 |
| SATB1 | miR-3926 | Predicted | >90 |
| CCER1 | miR-3926 | Predicted | >90 |
| ATP11A | miR-3926 | Predicted | >90 |
| RBMX | miR-3926 | Predicted | >90 |
| HCFC2 | miR-3926 | Predicted | >90 |
| EEF1A1 | miR-3926 | Predicted | >90 |
| CEP350 | miR-3926 | Predicted | >90 |
| NCMAP | miR-3926 | Predicted | >90 |
| PIGO | miR-640 | Validated | NA |
| ARHGEF39 | miR-640 | Validated | NA |
| MELK | miR-640 | Validated | NA |
| FXN | miR-640 | Validated | NA |
| S1PR3 | miR-640 | Validated | NA |
| C9orf3 | miR-640 | Validated | NA |
| MSANTD3 | miR-640 | Validated | NA |
| JPH2 | miR-640 | Validated | NA |
| TLR6 | miR-640 | Validated | NA |
| LIAS | miR-640 | Validated | NA |
| ZBTB43 | miR-640 | Validated | NA |
| HFE | miR-640 | Validated | NA |
| WDR12 | miR-640 | Validated | NA |
| CYP20A1 | miR-640 | Validated | NA |
| ARPC2 | miR-640 | Validated | NA |
| C2orf72 | miR-640 | Validated | NA |
| CYTIP | miR-640 | Validated | NA |
| SLCO2B1 | miR-640 | Validated | NA |
| MPPE1 | miR-640 | Validated | NA |
| ZNF519 | miR-640 | Validated | NA |
| LIPG | miR-640 | Validated | NA |
| EIF5 | miR-640 | Validated | NA |
| APOPT1 | miR-640 | Validated | NA |
| IVD | miR-640 | Validated | NA |
| TMOD2 | miR-640 | Validated | NA |
| SOCS7 | miR-640 | Validated | NA |
| FBLIM1 | miR-640 | Validated | NA |
| PPP1R3B | miR-640 | Validated | NA |
| GSR | miR-640 | Validated | NA |
| LACTB | miR-640 | Validated | NA |
| STOML1 | miR-640 | Validated | NA |
| ZSCAN2 | miR-640 | Validated | NA |
| SLC28A1 | miR-640 | Validated | NA |
| MFSD11 | miR-640 | Validated | NA |
| APEX2 | miR-640 | Validated | NA |
| TIRAP | miR-640 | Validated | NA |
| ALDOA | miR-640 | Validated | NA |
| TRIM72 | miR-640 | Validated | NA |
| TTC31 | miR-640 | Validated | NA |
| CHCHD5 | miR-640 | Validated | NA |
| MAN1A2 | miR-640 | Validated | NA |
| RNF115 | miR-640 | Validated | NA |
| ENSA | miR-640 | Validated | NA |
| CWF19L1 | miR-640 | Validated | NA |
| HIF1AN | miR-640 | Validated | NA |
| AS3MT | miR-640 | Validated | NA |
| LRRC27 | miR-640 | Validated | NA |
| ZNF84 | miR-640 | Validated | NA |
| DIS3 | miR-640 | Validated | NA |
| TIMM50 | miR-640 | Validated | NA |
| C19orf47 | miR-640 | Validated | NA |
| ZNF576 | miR-640 | Validated | NA |
| RTN2 | miR-640 | Validated | NA |
| OPA3 | miR-640 | Validated | NA |
| SLC1A5 | miR-640 | Validated | NA |
| FUT2 | miR-640 | Validated | NA |
| FLVCR1 | miR-640 | Validated | NA |
| DEGS1 | miR-640 | Validated | NA |
| CHST6 | miR-640 | Validated | NA |
| CRK | miR-640 | Validated | NA |
| XPOT | miR-640 | Validated | NA |
| DCUN1D2 | miR-640 | Validated | NA |
| POTEM | miR-640 | Validated | NA |
| NUBPL | miR-640 | Validated | NA |
| POLM | miR-640 | Validated | NA |
| HIP1 | miR-640 | Validated | NA |
| GATAD1 | miR-640 | Validated | NA |
| EIF2A | miR-640 | Validated | NA |
| XIAP | miR-640 | Validated | NA |
| APOBEC3A | miR-640 | Validated | NA |
| FAM9B | miR-640 | Validated | NA |
| BCLAF3 | miR-640 | Validated | NA |
| USP9X | miR-640 | Validated | NA |
| HAS2 | miR-640 | Validated | NA |
| ZNF34 | miR-640 | Validated | NA |
| FPR2 | miR-640 | Validated | NA |
| ZNF347 | miR-640 | Validated | NA |
| CACNG8 | miR-640 | Validated | NA |
| ZNF584 | miR-640 | Validated | NA |
| RTL10 | miR-640 | Validated | NA |
| YPEL1 | miR-640 | Validated | NA |
| ITIH5 | miR-640 | Validated | NA |
| DHTKD1 | miR-640 | Validated | NA |
| CACNB2 | miR-640 | Validated | NA |
| RNF11 | miR-640 | Validated | NA |
| TTC4 | miR-640 | Validated | NA |
| IL23R | miR-640 | Validated | NA |
| GBP4 | miR-640 | Validated | NA |
| BORCS5 | miR-640 | Validated | NA |
| ASB8 | miR-640 | Validated | NA |
| CMBL | miR-640 | Validated | NA |
| TNPO1 | miR-640 | Validated | NA |
| F2R | miR-640 | Validated | NA |
| F2RL1 | miR-640 | Validated | NA |
| DARS2 | miR-640 | Validated | NA |
| PHLDA3 | miR-640 | Validated | NA |
| KLHDC8A | miR-640 | Validated | NA |
| MAP3K9 | miR-640 | Validated | NA |
| EFCAB11 | miR-640 | Validated | NA |
| TRIP11 | miR-640 | Validated | NA |
| RAB11FIP1 | miR-640 | Validated | NA |
| PLEKHA2 | miR-640 | Validated | NA |
| HOOK3 | miR-640 | Validated | NA |
| PDE7A | miR-640 | Validated | NA |
| TSPYL1 | miR-640 | Validated | NA |
| PBOV1 | miR-640 | Validated | NA |
| ZBTB8B | miR-640 | Validated | NA |
| RBBP4 | miR-640 | Validated | NA |
| RNF19B | miR-640 | Validated | NA |
| AGO3 | miR-640 | Validated | NA |
| CCDC30 | miR-640 | Validated | NA |
| GLTP | miR-640 | Validated | NA |
| ALDH2 | miR-640 | Validated | NA |
| P2RX7 | miR-640 | Validated | NA |
| SLC35F6 | miR-640 | Validated | NA |
| WDR92 | miR-640 | Validated | NA |
| ORAI2 | miR-640 | Validated | NA |
| HILPDA | miR-640 | Validated | NA |
| UBN2 | miR-640 | Validated | NA |
| NWD1 | miR-640 | Validated | NA |
| ZNF430 | miR-640 | Validated | NA |
| ZNF431 | miR-640 | Validated | NA |
| ZNF708 | miR-640 | Validated | NA |
| ZNF43 | miR-640 | Validated | NA |
| ZNF257 | miR-640 | Validated | NA |
| ZNF724 | miR-640 | Validated | NA |
| NPHS1 | miR-640 | Validated | NA |
| COX8A | miR-640 | Validated | NA |
| MRPL49 | miR-640 | Validated | NA |
| METTL14 | miR-640 | Validated | NA |
| SEC24D | miR-640 | Validated | NA |
| TMEM154 | miR-640 | Validated | NA |
| PDLIM3 | miR-640 | Validated | NA |
| PDE6A | miR-640 | Validated | NA |
| CCDC69 | miR-640 | Validated | NA |
| KCNMB1 | miR-640 | Validated | NA |
| STK25 | miR-640 | Validated | NA |
| DCP2 | miR-640 | Validated | NA |
| SNX2 | miR-640 | Validated | NA |
| IRF1 | miR-640 | Validated | NA |
| MAN2A2 | miR-640 | Validated | NA |
| CLUAP1 | miR-640 | Validated | NA |
| EMP2 | miR-640 | Validated | NA |
| CD96 | miR-640 | Validated | NA |
| LRRC58 | miR-640 | Validated | NA |
| SLC15A2 | miR-640 | Validated | NA |
| TMCC1 | miR-640 | Validated | NA |
| MTRNR2L5 | miR-640 | Validated | NA |
| POLR3A | miR-640 | Validated | NA |
| FFAR4 | miR-640 | Validated | NA |
| KIF1C | miR-640 | Validated | NA |
| TRPV2 | miR-640 | Validated | NA |
| ABHD15 | miR-640 | Validated | NA |
| ZNF554 | miR-640 | Validated | NA |
| ATCAY | miR-640 | Validated | NA |
| ZNF426 | miR-640 | Validated | NA |
| TIMM29 | miR-640 | Validated | NA |
| SWSAP1 | miR-640 | Validated | NA |
| MRPS10 | miR-640 | Validated | NA |
| SENP6 | miR-640 | Validated | NA |
| FAM46A | miR-640 | Validated | NA |
| CNBP | miR-4767 | Validated | NA |
| TMUB1 | miR-4767 | Validated | NA |
| YIF1B | miR-4767 | Validated | NA |
| NICN1 | miR-4767 | Validated | NA |
| CPLX1 | miR-4767 | Validated | NA |
| HEXA | miR-4767 | Validated | NA |
| BBC3 | miR-4767 | Validated | NA |
| ZNF107 | miR-4767 | Validated | NA |
| TMEM120B | miR-4767 | Validated | NA |
| ZBTB3 | miR-4767 | Validated | NA |
| STC2 | miR-4767 | Validated | NA |
| BAG5 | miR-4767 | Validated | NA |
| BRSK2 | miR-4767 | Validated | NA |
| ZNF124 | miR-4767 | Validated | NA |
| TOX4 | miR-4767 | Validated | NA |
| PDE7A | miR-4767 | Validated | NA |
| SDF4 | miR-4767 | Validated | NA |
| DHX40 | miR-4767 | Validated | NA |
| REEP3 | miR-4767 | Validated | NA |
| STK11 | miR-4767 | Validated | NA |
| SNRPD1 | miR-4767 | Validated | NA |
| HLCS | miR-4767 | Validated | NA |
| SUGT1 | miR-4767 | Validated | NA |
| SRP54 | miR-4767 | Validated | NA |
| TXNDC16 | miR-4767 | Validated | NA |
| CHST12 | miR-4767 | Validated | NA |
| CPSF2 | miR-4767 | Validated | NA |
| PIAS4 | miR-4767 | Validated | NA |
| ZNF878 | miR-4767 | Validated | NA |
| ZNF451 | miR-4767 | Validated | NA |
| ZNF324B | miR-4767 | Validated | NA |
| NUMA1 | miR-4767 | Validated | NA |
| KIAA1551 | miR-4767 | Validated | NA |
| VPS51 | miR-4767 | Validated | NA |
| ZBTB22 | miR-4767 | Validated | NA |
| TNFSF15 | miR-4767 | Validated | NA |
| CDH7 | miR-4767 | Validated | NA |
| RAB11B | miR-4767 | Validated | NA |
| MELTF | miR-4767 | Validated | NA |
| CFAP97D1 | miR-4767 | Validated | NA |
| MAFK | miR-4767 | Validated | NA |
| S1PR2 | miR-4767 | Validated | NA |
| DIPK2A | miR-3156-5p | Predicted | >90 |
| CDC123 | miR-3156-5p | Predicted | >90 |
| SESTD1 | miR-3156-5p | Predicted | >90 |
| C3orf38 | miR-3156-5p | Predicted | >90 |
| PLAG1 | miR-3156-5p | Predicted | >90 |
| KLHL11 | miR-3156-5p | Predicted | >90 |
| KCNJ16 | miR-3156-5p | Predicted | >90 |
| PML | miR-3156-5p | Predicted | >90 |
| POLR3G | miR-3156-5p | Predicted | >90 |
| RAP1A | miR-3156-5p | Predicted | >90 |
| CHAC2 | miR-3156-5p | Predicted | >90 |
| RAB27A | miR-3156-5p | Predicted | >90 |
| ROCK2 | miR-3156-5p | Predicted | >90 |
| ZNF273 | miR-3156-5p | Predicted | >90 |
| BCAT1 | miR-3156-5p | Predicted | >90 |
| XRN1 | miR-3156-5p | Predicted | >90 |
| TACC1 | miR-3156-5p | Predicted | >90 |
| LYZ | miR-3156-5p | Predicted | >90 |
| SDC2 | miR-3156-5p | Predicted | >90 |
| NIPAL2 | miR-3156-5p | Predicted | >90 |
| WDR33 | miR-5699 | Validated | NA |
| RBM14 | miR-5699 | Validated | NA |
| CCND1 | miR-5699 | Validated | NA |
| G6PC | miR-5699 | Validated | NA |
| PLEKHG5 | miR-5699 | Validated | NA |
| GALNT11 | miR-5699 | Validated | NA |
| DYNLL2 | miR-5699 | Validated | NA |
| H3F3B | miR-5699 | Validated | NA |
| CENPI | miR-5699 | Validated | NA |
| SHOC2 | miR-5699 | Validated | NA |
| CEACAM19 | miR-5699 | Validated | NA |
| CES3 | miR-5699 | Validated | NA |
| VPS4A | miR-5699 | Validated | NA |
| GLG1 | miR-5699 | Validated | NA |
| KLHDC4 | miR-5699 | Validated | NA |
| NPC1L1 | miR-5699 | Validated | NA |
| EIF4G1 | miR-5699 | Validated | NA |
| COL14A1 | miR-5699 | Validated | NA |
| ISG20L2 | miR-5699 | Validated | NA |
| ZDHHC8 | miR-5699 | Validated | NA |
| LZTR1 | miR-5699 | Validated | NA |
| DDTL | miR-5699 | Validated | NA |
| SAPCD2 | miR-5699 | Validated | NA |
| MFAP5 | miR-5699 | Validated | NA |
| RIMKLB | miR-5699 | Validated | NA |
| GRIN2B | miR-5699 | Validated | NA |
| KMT2D | miR-5699 | Validated | NA |
| PLEKHA6 | miR-5699 | Validated | NA |
| GTF2A1 | miR-5699 | Validated | NA |
| KCNK10 | miR-5699 | Validated | NA |
| HPSE | miR-5699 | Validated | NA |
| NUP205 | miR-5699 | Validated | NA |
| FADS1 | miR-5699 | Validated | NA |
| PEX16 | miR-5699 | Validated | NA |
| POLR3G | miR-5699 | Validated | NA |
| TSLP | miR-5699 | Validated | NA |
| NHLRC4 | miR-5699 | Validated | NA |
| ARHGAP31 | miR-5699 | Validated | NA |
| PPP3CB | miR-5699 | Validated | NA |
| GHITM | miR-5699 | Validated | NA |
| CNTROB | miR-5699 | Validated | NA |
| PER1 | miR-5699 | Validated | NA |
| TVP23C | miR-5699 | Validated | NA |
| TVP23B | miR-5699 | Validated | NA |
| PIAS4 | miR-5699 | Validated | NA |
| ELAVL3 | miR-5699 | Validated | NA |
| MDGA1 | miR-5699 | Validated | NA |
| ATPAF1 | miR-4300 | Predicted | >90 |
| TNRC6B | miR-4300 | Predicted | >90 |
| PLXNA4 | miR-4300 | Predicted | >90 |
| NOA1 | miR-4300 | Predicted | >90 |
| SMCR8 | miR-4300 | Predicted | >90 |
| GINS2 | miR-4300 | Predicted | >90 |
| RASGRP3 | miR-4300 | Predicted | >90 |
| PRX | miR-4300 | Predicted | >90 |
| LY6G6C | miR-4300 | Predicted | >90 |
| NUCB2 | miR-4300 | Predicted | >90 |
| MYCT1 | miR-4300 | Predicted | >90 |
| HIC1 | miR-4300 | Predicted | >90 |
| EDA2R | miR-4300 | Predicted | >90 |
| DUSP23 | miR-4300 | Predicted | >90 |
| FBXO10 | miR-4300 | Predicted | >90 |
| CLEC4E | miR-4300 | Predicted | >90 |
| TGFBR1 | miR-3156-5p | Validated | NA |
| SLC44A1 | miR-3156-5p | Validated | NA |
| RBM12 | miR-3156-5p | Validated | NA |
| SVEP1 | miR-3156-5p | Validated | NA |
| E2F3 | miR-3156-5p | Validated | NA |
| NBEAL1 | miR-3156-5p | Validated | NA |
| HOXD9 | miR-3156-5p | Validated | NA |
| TXNL1 | miR-3156-5p | Validated | NA |
| FGF7 | miR-3156-5p | Validated | NA |
| TLK2 | miR-3156-5p | Validated | NA |
| ITPRIPL2 | miR-3156-5p | Validated | NA |
| ATXN2L | miR-3156-5p | Validated | NA |
| SPNS1 | miR-3156-5p | Validated | NA |
| U2SURP | miR-3156-5p | Validated | NA |
| SMC4 | miR-3156-5p | Validated | NA |
| KLHL15 | miR-3156-5p | Validated | NA |
| MAL2 | miR-3156-5p | Validated | NA |
| TG | miR-3156-5p | Validated | NA |
| IGSF9 | miR-3156-5p | Validated | NA |
| VANGL2 | miR-3156-5p | Validated | NA |
| SNAP29 | miR-3156-5p | Validated | NA |
| SUV39H2 | miR-3156-5p | Validated | NA |
| UGT3A1 | miR-3156-5p | Validated | NA |
| RABGAP1L | miR-3156-5p | Validated | NA |
| FAM20B | miR-3156-5p | Validated | NA |
| YOD1 | miR-3156-5p | Validated | NA |
| SMOC2 | miR-3156-5p | Validated | NA |
| SLC26A7 | miR-3156-5p | Validated | NA |
| SDAD1 | miR-3156-5p | Validated | NA |
| ENPP1 | miR-3156-5p | Validated | NA |
| SLC5A8 | miR-3156-5p | Validated | NA |
| TMEM209 | miR-3156-5p | Validated | NA |
| METTL14 | miR-3156-5p | Validated | NA |
| KCNMB1 | miR-3156-5p | Validated | NA |
| ARL10 | miR-3156-5p | Validated | NA |
| CANX | miR-3156-5p | Validated | NA |
| TRIM71 | miR-3156-5p | Validated | NA |
| PCSK1 | miR-3156-5p | Validated | NA |
| CEP120 | miR-3156-5p | Validated | NA |
| AFF4 | miR-3156-5p | Validated | NA |
| HNRNPA0 | miR-3156-5p | Validated | NA |
| CD86 | miR-3156-5p | Validated | NA |
| ARID5B | miR-3156-5p | Validated | NA |
| MYPN | miR-3156-5p | Validated | NA |
| SAR1A | miR-3156-5p | Validated | NA |
| PPIF | miR-3156-5p | Validated | NA |
| SPC24 | miR-3156-5p | Validated | NA |
| SRPK1 | miR-3156-5p | Validated | NA |
| ZFP91 | miR-640 | Predicted | >90 |
| IGF2BP3 | miR-640 | Predicted | >90 |
| PUS10 | miR-640 | Predicted | >90 |
| TM9SF3 | miR-640 | Predicted | >90 |
| TNPO1 | miR-640 | Predicted | >90 |
| ABI3BP | miR-640 | Predicted | >90 |
| NELL1 | miR-640 | Predicted | >90 |
| CCNG1 | miR-640 | Predicted | >90 |
| ZBTB24 | miR-640 | Predicted | >90 |
| DMAC1 | miR-640 | Predicted | >90 |
| LSM12 | miR-640 | Predicted | >90 |
| GIGYF2 | miR-640 | Predicted | >90 |
| TADA2B | miR-640 | Predicted | >90 |
| HNRNPH3 | miR-640 | Predicted | >90 |

The comprehensive list of the validated and putative miRNA target genes is provided.
